# Can Glycerol Be Used as an Effective Alternative Storage Medium for Avulsed Teeth Before Replantation?

**DOI:** 10.7759/cureus.89336

**Published:** 2025-08-04

**Authors:** Saumya Taneja, Rajeev K Singh, Khushtar Haider, Prateek Shakti, Sweta Shabnam, Rakesh Kumar, Abbas A Mahdi, Gulshan Singh

**Affiliations:** 1 Department of Pedodontics and Preventive Dentistry, Inderprastha Dental College & Hospital, Ghaziabad, IND; 2 Department of Pediatric and Preventive Dentistry, King George's Medical University, Lucknow, IND; 3 Department of Dentistry, Maharani Laxmi Bai Medical College, Jhansi, IND; 4 Department of Orthodontics and Dentofacial Orthopaedics, All India Institute of Medical Sciences, Central Armed Police Forces Institute of Medical Sciences (CAPFIMS), New Delhi, IND; 5 Department of Biochemistry, Era University, Lucknow, IND; 6 Department of Orthodontics and Dentofacial Orthopaedics, King George's Medical University, Lucknow, IND

**Keywords:** glycerol, hbss, periodontal ligament cells, replantation, storage medium

## Abstract

Introduction

For successful replantation of avulsed teeth, preserving the vitality of periodontal ligament (PDL) cells of the avulsed tooth is important. The aim of the present study was to evaluate the usage of glycerol by comparing its different concentrations with the gold standard medium, i.e., Hank’s balanced salt solution (HBSS). Glycerol is a cryoprotective agent used in many cell preservation studies, but has never been used earlier as a storage medium for avulsed human teeth.

Methods

PDL cells were obtained from healthy human teeth extracted for orthodontic purposes from children aged eight to 15 years old and cultured in minimum essential medium (MEM). The cultures were exposed for one, six, 12, and 24 hours to various experimental solutions at 37°C. The data obtained were statistically analyzed using one-way ANOVA and post hoc Tukey test.

Results

The mean viability of HBSS was the highest amongst the three experimental solutions, followed by diluted 10% glycerol, and least in pure glycerol.

Conclusion

Preparation of 10% glycerol was effective as a short-term storage medium. We recommend the formulation of various concentrations of glycerol and determining its clinical effectiveness.

## Introduction

Modern dental practice is focused on the preservation of the natural tooth rather than tooth removal treatment options. Nowadays, it is widely accepted that there is nothing better than saving a natural tooth, and it should always be the first choice for the best functional and cosmetic results. Prosthetic replacement of teeth does not yield the same results as natural teeth are a part of a complex system of nerves, vessels, and other tissues in the oral cavity. Considering this, replantation of the tooth into the socket is considered to be the best treatment modality for cases of tooth avulsion [1].

Avulsion accounts for 1-11% of all traumatic injuries of permanent teeth [2]. For successful replantation of avulsed teeth, preserving the vitality of periodontal ligament (PDL) cells of the avulsed tooth is of paramount importance, which makes reattachment of PDL possible. Extra-alveolar dry time should not be more than five minutes for the best prognosis [3]. In cases of avulsion, either the tooth can be immediately replanted or it should be stored in an appropriate storage medium and transported to a dental surgeon for replantation. An ideal storage medium, along with having essential nutrients, physiologic osmolality, neutral pH, and clonogenic capacity to preserve the viability of PDL cells, should also be easily accessible [4,5].

Innumerable media have been experimented with for storing an avulsed tooth. These include tap water, saliva, saline, milk, Hank’s balanced salt solution (HBSS), coconut water, Viaspan, propolis, green tea extract (GTE), egg albumin, and Custodiol [4-6]. HBSS is considered the gold standard storage medium, as recommended by the International Association of Dental Traumatology, as it is non-toxic, pH-balanced, and contains many essential nutrients [7]. The osmolality and pH of HBSS are 270-290 mosmol/kg and 7.2, respectively. The only shortcoming of this synthetic medium is its unavailability at the accident site [8]. Hence, there is always a never-ending quest for a substitute that can be easily available.

Recently, many natural products like coconut water, propolis, egg albumin, GTE, and others have been investigated for preserving the vitality of PDL cells, but none of them have proved to be completely effective. In the quest for finding a more readily available storage medium, we have designed this study to evaluate the efficacy of glycerol as a storage medium. Glycerol is used as a cryoprotective agent in preserving PDL of monkey teeth, but has never been used as a storage medium for avulsed human teeth. Hence, this study was conducted to evaluate the usage of glycerol by comparing its different concentrations with the gold standard synthetic storage medium HBSS on the parameters of optical density and viability of cells.

## Materials and methods

The study was conducted after approval of King George's Medical University Institutional Ethics Committee (69 ECM IIB/P37) from April 2014 to June 2015. Forty orthodontically extracted healthy teeth of children from the age of eight to 15 years were collected immediately following extraction and were washed with sterile normal saline to remove the residual blood. The extracted tooth was stored in a 15 ml sterile Falcon tube containing minimum essential medium (MEM).

After rinsing the tooth with phosphate buffer saline (PBS) multiple times, the PDL tissue was scraped from the middle third of the root. PDL cells were placed in tissue culture Petri dishes supplemented with MEM containing 14% fetal bovine serum (FBS) and 1% of antibiotic/antimycotic solution and incubated at 37°C in an atmosphere of 5% CO2. A total of 40 teeth from different individuals were studied, out of which cultures for 30 teeth were successfully established.

The three media that were compared were HBSS, pure glycerol, and 10% glycerol. HBSS was used in its directly available form. Pure glycerol has never been used before as a storage medium for avulsed teeth. However, we have used it to evaluate its potential without dilution, as glycerol will only be available to the general public in its pure form in the market. Glycerol was obtained from the market and then sterilized by passing through a 0.22 µm filter and stored in 100 ml aliquots at 4°C. A solution of 10% glycerol was prepared by adding one volume of glycerol to nine volumes of ultra-pure water to obtain 10% glycerol. The solution was then sterilized by passing through a 0.22 µm filter and stored in 100 ml aliquots at 4°C.

Once 80% confluency of cells was reached, 12 × 103 PDL cells were plated in 96-well culture plates, followed by incubation at 37°C with 5% CO2 and 95% air overnight. MEM was then removed, and the wells were filled with 100 µL of each storage medium (HBSS, 100% glycerol, and 10% glycerol) for 24 hours. The viability of cells was recorded at one, six, 12, and 24 hours. MTT (3-(4,5-dimethylthiazol-2-yl)-2,5-diphenyltetrazolium bromide) was added (10 µl per well of 5 mg/ml stock solution) at each time interval, followed by incubation for three hours. Dimethyl sulfoxide (DMSO) was added (200 µl) in each well after removing MEM for solubilization of formazan. Absorbance was observed after 10 minutes at 550 nm using a multimode microplate reader (Synergy HT, Biotek, Winooski, VT). The optical density (OD) was measured by means of a spectrophotometer at a 550 nm wavelength, which represented the viability of metabolically active cells. Cell morphology was also evaluated by phase contrast microscopy under a 20x objective. The observations were recorded and subjected to statistical analysis.

Statistical analysis

Continuous data were summarized as mean ± standard deviation (SD). The independent groups were compared by one-way analysis of variance (ANOVA), and the significance of the mean difference between the groups was determined by the post hoc Tukey test. A two-tailed (α = 2) p-value less than 0.05 (p < 0.05) was considered statistically significant. Analysis was performed using GraphPad Prism software version 5.0 (GraphPad Software, San Diego, CA).

## Results

**Figure 1:**
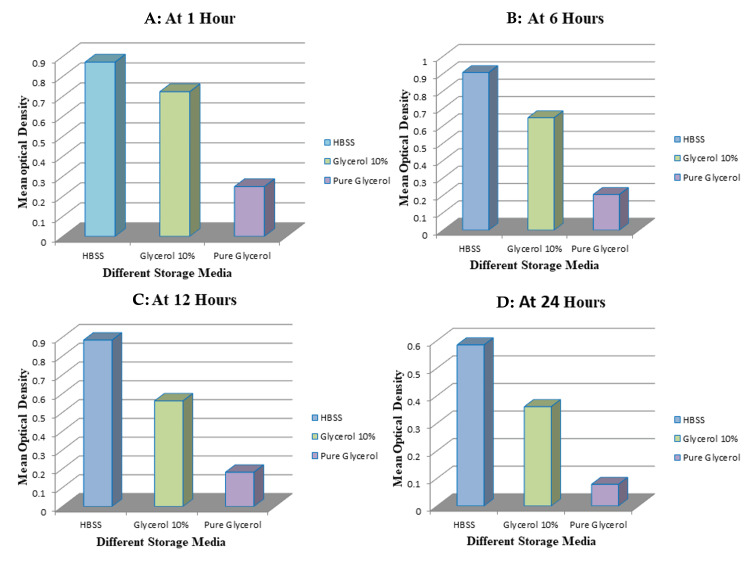
Optical density of various storage media. A: At one hour. B: At six hours. C: At 12 hours. D: At 24 hours. HBSS: Hank’s balanced salt solution.

The viability of PDL cells was assessed by measuring the OD of cells stored in three different media (HBSS, 10% glycerol (GLY10), and pure glycerol (GLYP)) at intervals of one, six, 12, and 24 hours. The OD, indicative of cell viability, was consistently highest in the HBSS group across all time points, followed by 10% glycerol, with the lowest values recorded in pure glycerol. At one hour, cells stored in HBSS showed the highest viability (OD = 0.871 ± 0.082), followed by 10% glycerol (0.724 ± 0.134), while pure glycerol showed markedly lower viability (0.249 ± 0.073) (Figure 1A).

At the six-hour mark, the OD of cells preserved in HBSS peaked at 0.90675 ± 0.052, suggesting maximum cellular viability. In contrast, 10% glycerol showed a moderate OD of 0.64650 ± 0.071, while pure glycerol presented a markedly reduced OD of 0.20500 ± 0.018, reflecting significant cellular deterioration (Figure 1B).

By 12 hours, although all three groups demonstrated a decline in OD, the reduction was not statistically significant. HBSS continued to maintain the highest OD (0.88525 ± 0.050), indicating its superior preservation capabilities, followed by 10% glycerol (0.56175 ± 0.076), and pure glycerol (0.18225 ± 0.071) (Figure 1C).

At the 24-hour interval, a substantial decrease in cell viability was observed across all media, reaching statistical significance (p < 0.005). Nonetheless, HBSS still demonstrated the best preservation with an OD of 0.58425 ± 0.024, which corresponds to roughly 81% cell viability. The 10% glycerol solution retained approximately 50% viable cells (OD = 0.36025 ± 0.123), whereas pure glycerol exhibited severe cytotoxicity, with the lowest viability (OD = 0.07850 ± 0.052). This steep decline in the GLYP group underscores the detrimental effect of undiluted glycerol on cell survival (Figure 1D).

The findings summarized in Figure 1 and Table 1 clearly illustrate the time-dependent decline in cell viability and highlight HBSS as the most effective storage medium among those tested.

**Table 1:** Intragroup comparison of optical density (mean ± SD) of different storage media at different intervals. HBSS: Hank’s balanced salt solution; GLY10: glycerol 10%; GLYP: glycerol pure; SD: standard deviation. P < 0.005 is considered significant.

Groups	1 hour	6 hours	12 hours	24 hours	F value	p-value
HBSS	0.87175 ± 0.082	0.90675 ± 0.052	0.88525 ± 0.050	0.58425 ± 0.024	29.389	0.000
GLY10	0.72375 ± 0.134	0.64650 ± 0.071	0.56175 ± 0.076	0.36025 ± 0.123	8.271	0.002
GLYP	0.24925 ± 0.073	0.20500 ± 0.018	0.18225 ± 0.071	0.07850 ± 0.052	6.263	0.008

**Figure 2:**
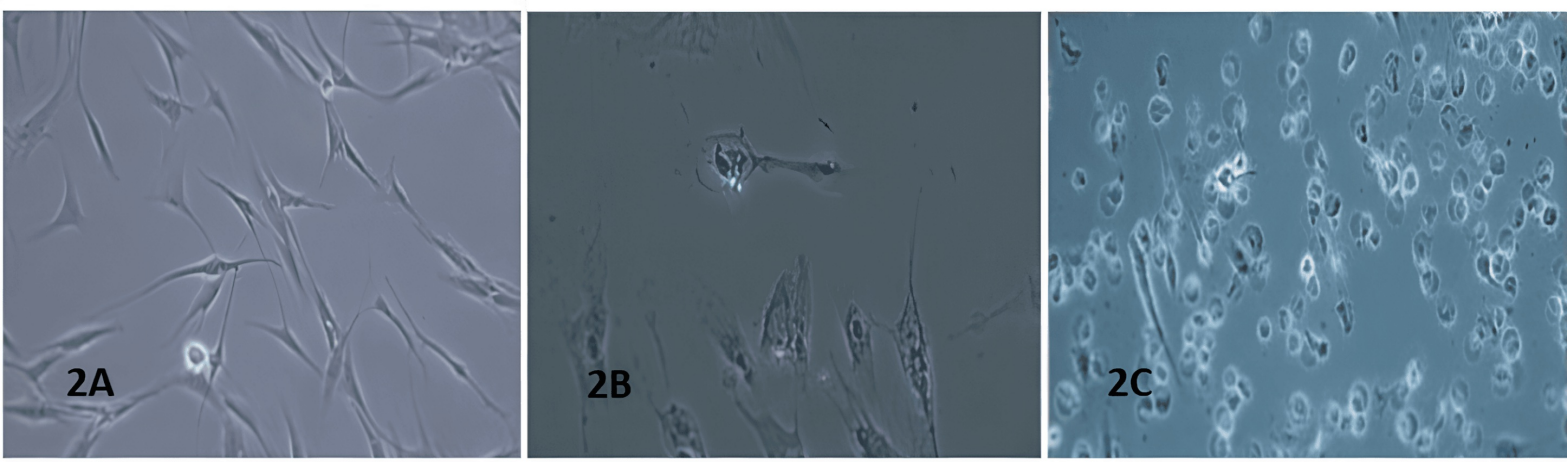
Microscopic analysis of periodontal ligament (PDL) cells in different storage media at 24 hours. A: Hank’s balanced salt solution. B: 10% glycerol. C: Pure glycerol.

Microscopic evaluation under 20x magnification further corroborated the OD findings. As shown in Figure 2A, PDL cells stored in HBSS retained their spindle-shaped morphology even after 24 hours, suggesting minimal cellular damage. Conversely, cells preserved in 10% glycerol displayed morphological alterations and signs of apoptosis by the 24-hour point (Figure 2B). The most severe morphological degeneration was observed in cells exposed to pure glycerol (Figure 2C), where cellular fixation and shrinkage were noted, likely due to the medium’s high viscosity and hypertonic nature. Osmotic imbalance may have caused excessive water efflux from the cells, leading to extensive cell death in the pure glycerol group.

In conclusion, HBSS emerged as the most biocompatible and effective medium for maintaining PDL cell viability, followed by 10% glycerol, while pure glycerol was found to be unsuitable due to its cytotoxic effects over time.

## Discussion

Tooth avulsion, which signifies complete dislodgment of the tooth from its socket, is a usual consequence following orofacial trauma. The prognosis of an avulsed tooth depends upon the measures taken by the patient and clinician immediately post avulsion. The treatment of choice for an avulsed tooth is immediate replantation, which may not always be possible after trauma due to a lack of expertise of people at the trauma site or other grievous injuries to the patient. The probability of survival of a replanted tooth depends on the viability of PDL cells attached to the tooth [9]. Extra-oral dry time and suitability of storage medium are the critical factors that determine the volume of viable PDL cells on the avulsed tooth [10]. If these factors are not taken into consideration, the chances of inflammatory and replacement resorption increase, leading to failure of replanted avulsed tooth [11-13]. Hence, the availability of a suitable medium at the trauma site is beneficial for replantation to be successful.

HBSS has been recommended as the gold standard storage medium for avulsed teeth by the International Association of Dental Traumatology, but no consensus exists on its ability to preserve the viability of PDL cells [10]. Its application is also restricted to hospitals or laboratories. Its unavailability at trauma sites and high cost make it an impractical storage medium for avulsed teeth [8].

Glycerol, used as an experimental storage medium in the present study, on the other hand, is easily available and inexpensive. Glycerol has been studied for the preservation of monkey teeth and for tooth germs as well in the past [14]. Glycerol is basically a polyol and cryoprotective agent, compounds of which are used as preservatives in lubricants, lotions, inks, and fruits [15,16]. It is a nontoxic, sweet-tasting, viscous fluid with a pH of 6.9-7.7, which is favorable for preservation of PDL cells. It is a known lubricant or humectant having bacteriostatic properties, and used globally in personal care, pharmaceuticals, food & beverages, tobacco, alkyd resins, polyether, polyols, and preserving agent in botanical extracts. In a study conducted by Zhang et al., glycerol used as a transport and storage medium for swab samples of animals for the detection of avian influenza virus proved to be a better medium than the rest [17]. Glycerol has also been seen to increase the survival rate of cultured epithelial sheets [18]. Pig skin allografts can be successfully stored when preserved in 85% glycerol [19,20]. Irradiated sterilized gelatin-water-glycerol gel proved to be useful as an injectable carrier for bone tissue engineering [21]. However, its use as a storage medium for PDL cells has not been established. Hence, in the present study, we used glycerol to evaluate its potential as a storage medium because of its better availability compared to HBSS and the known healing, nutritive, and bacteriostatic properties, which it possesses for human tissues, especially in skin care.

The present study revealed that the teeth in HBSS medium demonstrated the highest number of mean viable cells, followed in order by glycerol 10% and pure glycerol. The result of the effectiveness of HBSS is also in agreement with other studies for periods varying from three to 72 hours [11,22-27]. Glycerol in 10% concentration preserved the viability of 72% cells at one hour and almost 56% cells after 12 hours. Pure glycerol, on the other hand, preserved 25% viability at one hour, falling to 7% at the end of 24 hours. On analyzing the results, we can recommend the use of 10% glycerol as an effective short-term storage medium.

The method of primary cell culture suggested by Doyle et al. [28] and the cell line method are the two methods used for assessing the potency of the storage medium [29,30]. The cell line method was used in the present study, where the PDL cells are first isolated from the teeth and cultured, followed by assessment of viability by immersing in experimental storage medium. In the primary culture method, the viability of periodontal ligament cells isolated from the extracted teeth is used directly to assess the potency of each storage medium. The method used in the present study could be a confounding factor, as the cells cultured may contain more nutrients in comparison to the natural scenario post replantation.

Limitations and variabilities do exist in the present in vitro study design. The extractions were performed by different clinicians, inducing variable trauma to the PDL, inconsistent with the actual avulsion cases. The culture of PDL cells in vitro does not necessarily simulate the oral environment of the patients. Supplementation of culture media had an additive effect on the nutritional supply of fibroblasts. Hence, the clinical effectiveness of the experimental media needs to be substantiated by in vivo studies with a more standardized setting.

## Conclusions

From the present study, we can conclude that pure glycerol, which is easily available, did not show very good results. So, we do not recommend it as a preferred storage medium for long-term storage. Preparation of 10% glycerol was effective as a short-term storage medium, as 50% of cells remained viable even after 24 hours. We also recommend the formulation of various concentrations of glycerol and determining its clinical effectiveness by further in vivo experiments.
